# Towards a clinically useful diagnosis for mild-to-moderate conditions of medically unexplained symptoms in general practice: a mixed methods study

**DOI:** 10.1186/1471-2296-15-118

**Published:** 2014-06-12

**Authors:** Mette T Rask, Rikke S Andersen, Flemming Bro, Per Fink, Marianne Rosendal

**Affiliations:** 1 The Research Unit for General Practice, Section for General Practice, Department of Public Health, Aarhus University, Bartholins Allé 2, 8000 Aarhus C, Denmark; 2 The Research Clinic for Functional Disorders and Psychosomatics, Aarhus University Hospital, Barthsgade 5, 8200 Aarhus N, Denmark

**Keywords:** Somatoform disorders, Signs and symptoms, Classification, Diagnosis, Focus groups, General practice, Primary health care

## Abstract

**Background:**

Symptoms that cannot be attributed to any known conventionally defined disease are highly prevalent in general practice. Yet, only severe cases are captured by the current diagnostic classifications of medically unexplained symptoms (MUS). This study explores the clinical usefulness of a proposed new diagnostic category for mild-to-moderate conditions of MUS labelled ‘multiple symptoms’.

**Methods:**

A mixed methods approach was used. For two weeks, 20 general practitioners (GPs) classified symptoms presented in consecutive consultations according to the International Classification of Primary Care (ICPC) supplemented with the new diagnostic category ‘multiple symptoms’. The GPs’ experiences were subsequently explored by focus group interviews. Interview data were analysed according to ethnographic principles.

**Results:**

In 33% of patients, GPs classified symptoms as medically unexplained, but applied the category of ‘multiple symptoms’ only in 2.8%. The category was described as a useful tool for promoting communication and creating better awareness of patients with MUS; as such, the category was perceived to reduce the risk of unnecessary tests and referrals of these patients. Three main themes were found to affect the clinical usefulness of the diagnostic category of ‘multiple symptoms’: 1) lack of consensus on categorisation practices, 2) high complexity of patient cases and 3) relational continuity (i.e. continuity in the doctor-patient relationship over time). The first two were seen as barriers to usefulness, the latter as a prerequisite for application. The GPs’ diagnostic classifications were found to be informed by the GPs’ subjective pre-formed concepts of patients with MUS, which reflected more severe conditions than actually intended by the new category of ‘multiple symptoms’.

**Conclusions:**

The study demonstrated possible clinical benefits of the category of ‘multiple symptoms’, such as GPs’ increased awareness and informational continuity in partnership practices. The use of the category was challenged by the GPs’ conceptual understanding of MUS and was applied only to a minority of patients. The study demonstrates a need for addressing these issues if sub-threshold categories for MUS are to be applied in routine care. The category of ‘multiple symptoms’ may profitably be used in the future as a risk indicator rather than a diagnostic category.

## Background

Medically unexplained symptoms (MUS) or functional somatic symptoms are defined as somatic symptoms that cannot be attributed to any known, conventionally defined disease [1]. Patients with MUS are highly prevalent in general practice [2,3].

In the International Classification of Diseases (ICD-10) and the Diagnostic and Statistical Manual of Mental Disorders (DSM-IV), MUS are classified as somatoform disorders. Due to the restrictive criteria of these classifications, the categories of somatoform disorders capture only severe conditions and thus exclude the majority of patients with MUS encountered in general practice. Patients may accordingly be left undiagnosed or misclassified and at risk of iatrogenic harm due to unnecessary tests and treatments [4-6]. Although much research has been devoted to the development of improved diagnostic categories of somatoform disorders, less attention has been paid to patients presenting with mild-to-moderate conditions [7-9].

In the International Classification of Primary Care (ICPC), MUS not fulfilling criteria for a specific diagnosis of a disease or disorder are to be classified by purely descriptive symptom diagnoses [10]. However, as symptom diagnoses rest on single symptoms, they produce a fragmented symptom picture and conditions of MUS may inherently be at risk of neglect.

Improvements in the ICPC-2 have been suggested with a view to classifying mild-to-moderate conditions of MUS [6]. Rosendal et al. specified the criteria for a new symptom diagnosis, ‘multiple symptoms’. To avoid any premature conclusions of symptom aetiology or symptom explanation this new category was intended as a solely descriptive symptom diagnosis suggested to be included in the ICPC chapter for ‘General and unspecified health complaints and diagnoses’ [6]. However, the proposed diagnostic category has not yet been empirically evaluated. In the front line of the health care system, the general practitioner (GP) encounters all kinds of undifferentiated health problems and interpretation and categorisation of symptoms are inherent parts of daily clinical practice. For the GP, a diagnostic category serves as a decision node or a working diagnosis on which treatment, further investigations and conclusions on the absence of serious disease are based [11]. To be clinically useful, a diagnostic category must therefore provide a useable framework for both the interpretation and the management of the problems encountered [12,13].

Aiming to explore the clinical usefulness of the new diagnostic category of ‘multiple symptoms’, we operationalised the diagnostic criteria for mild-to-moderate conditions of MUS based on the proposal by Rosendal et al. The diagnostic criteria for ‘multiple symptoms’ were formulated as: 1) The patient must have had at least three symptoms, independently or concomitantly of one another, 2) The presented symptoms must not be attributable to a verifiable disease or disorder, and 3) The symptoms have been present within the past six months. As ‘multiple symptoms’ is a diagnostic category which is applied by the GP on the basis of the clinical encounter, the symptoms included in ‘multiple symptoms’ should be significant symptoms. This implies that the patient is seeking health care for these symptoms or is somehow affected by them, but the patient need not be impaired or disabled.

In this paper, we explore GPs' classification of MUS, their application of 'multiple symptoms' in everyday clinical practice and their perceptions of and experiences with this new category.

## Methods

A mixed methods approach was used. Questionnaire data on 20 GPs’ identification and classification of MUS were linked to data from focus group interviews. The study was approved by the Danish Data Protection Agency (Ref. no. 2008-41-2969). According to the Scientific Committee for the Central Denmark Region, the Biomedical Research Ethics Committee System Act did not apply to this study and did not need their approval. The study adhered to the RATS guidelines on qualitative research [14].

### Setting and subjects

All GPs in the North Denmark Region (n = 362) were invited to participate in the study. Another 27 GPs with residency in the Central Denmark Region, who were specially trained in the management of patients with MUS according to the Extended Reattribution and Management (TERM) Model [15], were invited in order to qualify and inspire the discussion in the focus groups.

Few GPs accepted to participate. Participants received a remuneration of EUR 480 for attending an introduction meeting and a focus group interview and additionally EUR 1.6 per completed questionnaire. During the introduction meeting, the GPs were introduced to the study aims and instructed in the identification of patients with MUS, and they were informed about the ICPC coding principles.

### Questionnaires

Participants identified and classified symptoms presented in face-to-face encounters with consecutive patients (age 18–65 years) for a ten-day period. For each encounter, GPs registered the symptoms presented by the patient in a one-page questionnaire inquiring about whether these symptoms were attributable to any conventionally defined disease or disorder or not, and the problem was classified (see Additional file 1). Patients fulfilling criteria for well-defined diseases/disorders or functional somatic syndromes included as specific disease diagnoses in the ICPC (e.g. irritable bowel syndrome) were to be classified as such. If the GP could establish no specific diagnosis, the symptoms were considered to be medically unexplained. MUS were classified according to the ICPC as: 1) single symptoms expected to be self-limiting or awaiting further assessment, 2) unwarranted fear of having a disease or disorder, 3) ‘multiple symptoms’ or 4) somatoform disorder. Patients with MUS could be labelled by only one of these four categories. Encounters with patients who were not listed with the participating practices and encounters about prophylaxis were excluded from the study. Moreover, patients were registered at their first encounter and only once during the registration period.

### Focus group interviews

After the registration period, the GPs participated in two-hour focus group interviews, each with 4–6 participants. The focus group interviews were moderated by MTR, who was assisted by one of the co-authors (MR and FB).

The focus group interviews were based on a topic guide. First, GPs’ perceptions of the characteristics of patients with ‘multiple symptoms’ and their distinction between this group of patients and patients assigned to other diagnostic categories of MUS were explored (e.g. ‘*What are the characteristics of patients categorised as having ‘multiple symptoms’ compared with patients classified with a somatoform disorder?*’). Second, the GPs’ experiences with the clinical usefulness of the new diagnostic category were addressed (e.g. ‘*How did the category ‘multiple symptoms’ contribute to the encounter?*’). Throughout the focus group interviews, GPs were urged to explore each other’s views and to describe patient cases and specific encounters to exemplify attitudes and beliefs, thereby providing the group with an opportunity to discuss these issues. Hence, we sought to avoid that participants exclusively engaged in intellectual considerations over the subject matter.

### Data analysis

Based on the questionnaire data, descriptive analyses of the GPs’ classification practices were performed. Proportions of patients with MUS and the diagnostic subcategories according to GPs are presented as medians with 25 and 75 percentiles (inter-quartile range (IQR)). Data were analysed using STATA version 11.

The interviews were digitally recorded and transcribed verbatim. The software package NVivo version 8 was used for coding, sorting and retrieving data. Ethnographic principles of analysis were followed. Hence, in order to allow for reflexivity, the analytic process was not a process undertaken separately from the literature reviewing, the interviewing, the coding, the interpreting and the writing processes [16]. The analysis was undertaken by three of the authors holding different academic backgrounds (Master of Health Science, anthropologist and GP). The analysis was carried out as four partly intertwined steps: First, the transcribed interviews were read repeatedly and written summaries were made to get an overall impression of the material and to provide a basis for reflection and early insights (MTR, RSA, MR). Second, meaningful text units were coded while staying close to the data (MTR). Third, central concepts and themes emerging from the data were discussed by the research group. Literature studies further developed these concepts and themes and inspired to the use of a theoretical framework adopted from the medical sociologist, Annemarie Jutel [13,17]. Jutel describes diagnosis as being both a category and a process. As a category, diagnosis is assigned to patterns of symptoms or complaints. As a process, diagnosis is characterised by the assessment of symptom presentations [13]. According to this framework, the application of ‘multiple symptoms’ is understood as the categorisation itself preceded by a process of symptom interpretation. This framework laid the ground for the fourth step of the analysis, in which a more systematic coding was conducted and main concepts and themes were established.

## Results

Twenty-two GPs agreed to participate of whom 68% (n = 15) were male, 59% (n = 13) were working in partnership practices and 73% (n = 16) were working in practices located in urban areas. Ten of the GPs were trained according to the TERM model. GPs’ mean age was 55 years (SD 6.8). Two GPs were excluded from the statistical analyses due to deviations from the consecutive registration procedure. Another two were absent from the focus group interviews.

### Classification of MUS

**Figure 1:**
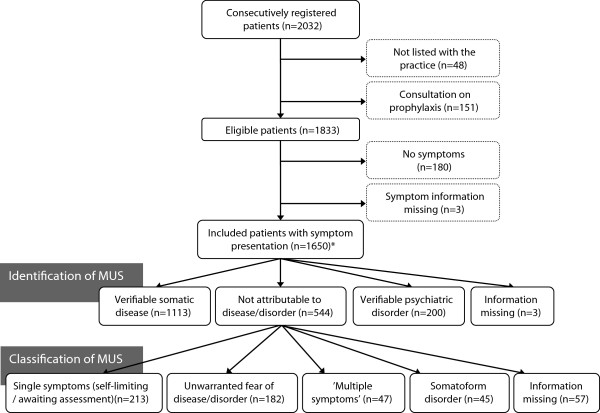
**Flow chart and questionnaire route.** *190 patients presented symptoms for which more than one possible reason was stated. The number of symptom explanations (somatic disease, psychiatric disorder and not attributable to any of these) therefore exceeds the number of included patients.

Twenty GPs included 1,650 patients with symptom presentation (Figure 1). Patients had a mean age of 44.3 years (SD 13.8) and 58.7% were female.In 33% (n = 544) of patients, GPs identified MUS, but only a minority was classified as ‘multiple symptoms’ (2.8%, n = 47) or somatoform disorder (2.7%, n = 45) (Figure 1). The GPs’ identification and classification of MUS varied substantially: the median for MUS was 22.0% (IQR: 17.3%-54.8%), the median for ‘multiple symptoms’ was 2.4% (IQR: 0.9%-4.4%) and the median for somatoform disorder was 1.6%, (IQR: 1.2%-4.4%). We found no statistically significant association between frequent use of the ‘multiple symptoms’ category and frequent use of the somatoform disorder category. TERM-trained GPs and non-TERM-trained GPs had comparable proportions of patients identified with MUS and categorised with ‘multiple symptoms’ and somatoform disorder (data not shown).

### Focus group interviews

By exploring the GPs’ perceptions of and experiences with the new diagnostic category of ‘multiple symptoms’, we identified three main themes of importance to its clinical usefulness: Categorisation of patients as having ‘multiple symptoms’ was hampered by (1) lack of consensus on categorisation practices and by (2) the complexity of the patient cases, whereas (3) relational continuity (i.e. continuity in the doctor-patient relationship over time) was disclosed as an essential prerequisite for the application of the diagnostic category of ‘multiple symptoms’.

#### ***Lack of consensus on categorisation practices***

In the focus group interviews, GPs were clear and unanimous about the theoretical boundaries between the four diagnostic categories for MUS, whereas boundaries became blurred in clinical case descriptions. Discrepancies between GPs illustrated that the process of categorising or diagnosing was not simply a formal process of deciphering symptoms through a lens of objective criteria, rather the process of diagnosing was equally an informal process influenced by GPs’ experiences and attitudes. In our material this became evident in subjective predefined concepts of patients with ‘multiple symptoms’.

*They are not suffering from the symptoms. They are not disability pensioners. They are living their lives and for periods of time they have this kind of reaction and they show up with these things.* (I03F01, no TERM-training)

*I think that these patients, to some extent, are suffering from a personality disorder. Thus, I’m not sure whether you can conclude that symptoms are not attributable to a psychiatric disorder.* (I05F02, TERM-training)

According to the GPs, patients with ‘multiple symptoms’ were difficult to distinguish, especially from patients presenting with unwarranted fear of a disease and patients with a somatoform disorder; and this difficulty meant that subjective and individual criteria were applied. The GPs’ distinction between patients with ‘multiple symptoms’ and patients with unwarranted fear depended on the ease with which patients could be reassured and the frequency of their visits. The extent of the patient’s impairment, health concerns and distress were additional factors that helped discriminate patients with a somatoform disorder from patients with ‘multiple symptoms’.

The subjective categorisation practices demonstrated that the GPs perceived the patients’ presentations of MUS to be of a more complex nature than reflected by the defining criteria of ‘multiple symptoms’, and hence this seems to demonstrate a discrepancy between the defining criteria of ‘multiple symptoms’ and the clinical experience. While the GPs’ subjective categorisation processes caused inconsistency in their application of ‘multiple symptoms’, their individual work with the diagnostic criteria made ‘multiple symptoms’ a tool for reflection and awareness that challenged their pre-existing perspectives.

*As a matter of fact, his wife has been ill. I’ve come to think whether this has been such a burden to him during the last couple of years that it’s now surfacing. I don’t know, but I’m certainly not done with it yet. […] Due to his personality, I didn’t even think of somatisation. Now, I suddenly realise that he fits this category very well.* (I13F03, TERM-training)

The heightened awareness of MUS was perceived as being instrumental in altering current practices because it gave the GP an opportunity to change patient management and thereby protect the patient from an endless odyssey in the healthcare system and possibly from development of a chronic condition of MUS.

#### ***Complexity of patient cases***

Diagnostic categories are supposed to reduce complexity. However, in our study the GPs did not find that the category of ‘multiple symptoms’ was helpful in reducing the complexity faced in the encounter with patients suffering from MUS.

An issue related to the difficulty embracing the identification and classification of MUS mentioned by the GPs in our study was that patients often present with a large variety of problems that may obscure symptom aetiology and make it difficult to rule out disease. The complexity of the patients’ complaints made the GPs fear misclassification and, not least, overlooking serious disease. Unless absolute certainty could be established in regard to ruling out serious disease, the GPs were reluctant to consider patient complaints as medically unexplained. Thus, the diagnostic category of ‘multiple symptoms’ was articulated as a diagnosis of exclusion although the GPs were aware of the dilemma of unnecessary and less reasonable referrals.

*I mean… it’s due to the fear of overlooking something… serious disease; but, on the other hand, we are offering them all sorts of referrals and all sorts of peculiar investigations because they are presenting these diffuse complaints. All the time we’re in this schism.* (I02F01, no TERM-training)

The GPs found the clinical context to be unsuited for addressing MUS. Time-restricted consultations made some of the GPs focus on what they considered more valid physical symptoms, thereby avoiding more complex and obligating issues in the clinical encounter.

*If the patient presents a symptom which seems valid, this is what I plunge into… because this is what I can cope with in 10 minutes, right […] If I apply a symptom diagnosis [‘multiple symptoms’], which I can’t immediately explain, then I have an obligation to continue, to explore it in depth, to do conversations and…well, maybe a raft of conversations, which I strictly speaking didn’t have the strength for or didn’t find the opportunity for that day.* (I10F02, TERM-training)

In our study, GPs expressed an obligation to take action if they applied the new symptom diagnosis. Established descriptive diagnostic categories for symptoms lacking a definite diagnosis do not offer a simple explanation or guide for treatment, nor does the diagnostic category of ‘multiple symptoms’. In our study, this caused uncertainty in the management of patients with ‘multiple symptoms’.

*I like to help people, so I prefer when they present something where I can explain what it is and what we’ll do. These patients, they become something, where I don’t know what to do, thus they are… they are not so pleasant to deal with.* (I11F03, no TERM-training)

Hence, the diagnostic category of ‘multiple symptoms’ was not perceived to bridge the gap between the identification of patients with MUS and the actions needed to help them.

#### ***Relational continuity***

GPs perceived relational continuity to be essential to the application of ‘multiple symptoms’. Familiarity with the patient was perceived to be helpful in the identification of symptom patterns, the interpretation of symptoms within a bio-psycho-social frame and in taking what was thought to be the appropriate action in the understanding of the patient’s personal and family background. The participating GPs felt confident that they could identify patients with MUS as soon as they crossed their door step. However, they were reluctant to apply the diagnosis of ‘multiple symptoms’ based on a single encounter and expressed a need for a course of events in order to err on the side of caution. Because of a perceived incapability of judging about the origin of symptoms, the GPs expressed reluctance towards the use of the diagnostic category of ‘multiple symptoms’ if they did not feel sufficiently familiar with the patient. On the other hand, if the GPs considered applying a diagnosis of somatoform disorder, a perceived lack of familiarity encouraged them to use the category of ‘multiple symptoms’ instead. Thus, careful not to err or to do wrong to the patient, the GPs chose the less stigmatising category.

*It is very objective/subjective… how well you know the patient before he or she is diagnosed with a somatoform disorder or whether you say: ‘what is this?’ I guess that is the way it is with diagnoses. You fear – in a way – to do wrong to the patient…* (I05F02, TERM-training)

Relational continuity is disrupted in partnership practices where patients consult more than one doctor, when doctors in training see the patient or when patients choose to be enlisted in another practice. In these cases, GPs perceived that the diagnostic category of ‘multiple symptoms’ could serve as a tool for communication.

*They are frequent visitors, and I think we overlook some of them, who maybe instead are being classified with single symptom diagnoses […] It would be a good way to inform each other that this is a patient with many symptoms and that we don’t have to refer her every single time she presents with a new symptom.* (I04F01, TERM-training)

In this way, ‘multiple symptoms’ was thought to support informational continuity by decreasing the inherent risk of overlooking patients with MUS due to single symptom diagnoses applied by different GPs and to hinder patients from undergoing futile referrals. However, the GPs were also aware of the risk of not taking symptoms seriously and of overlooking serious disease due to a stereotypical image of the patient based on familiarity with the patient over time or application of a label of ‘multiple symptoms’.

## Discussion

### Summary of main findings

To our knowledge, the present study is the first to address the use of a sub-threshold category for MUS from a clinician’s perspective. One third of GP face-to-face consultations were found to involve symptoms not attributable to a verifiable disease or disorder, yet the new diagnostic category of ‘multiple symptoms’ was applied in only 2.8% of the consultations. In focus group interviews, the GPs described ‘multiple symptoms’ as a useful tool for raising awareness of patients with MUS and for ensuring informational continuity if relational continuity was disrupted. Relational continuity was disclosed as an essential prerequisite for their interpretation of symptoms as medically unexplained and for the categorisation as ‘multiple symptoms’. We identified two main obstacles for using ‘multiple symptoms’ as a diagnostic category. First, there was a lack of consensus on the contents of the category of ‘multiple symptoms’. Second, the complexity of patient complaints challenged the application of this new diagnostic category, prompting the GPs to feel uncertain about how to manage patients assigned to this category.

### Strengths and limitations

The mixed methods approach was a major strength in the present study. As participating GPs practiced the classification of MUS during the study period, they were able to discuss hands-on experiences in the focus group interviews rather than just theorise about the new diagnostic category. Moreover, the combination of methods provided insight into the GPs’ actual behaviours and use of the new diagnostic category in clinical practice [18].

We included a sample of GPs representing both sexes and varying age, practice type and geographical location, which allowed us to obtain views across different demographic groups. The category of ‘multiple symptoms’ has not previously been applied in a clinical setting. Therefore, we wanted to obtain responses from GPs who were already trained in the management of patients with MUS and yet challenge potential conformity by also including non-trained GPs. The purposive sampling of GPs was a strength to the qualitative part of the study, but compromises the generalizability of our quantitative results. However, this was of minor concern as we intended not to extrapolate the quantitative findings, but to get insight into the GPs’ classification of MUS and to inspire the discussions in the focus group interviews.

While the group process is one of the major strengths of the focus group interview since this is where experiences and opinions are discussed and compared, this also holds a potential weakness as participants may modify their contribution to stick with the norm of the rest of the group [19]. This could pose a particular problem in this study, where GPs holding different qualifications in the management of patients with MUS were included. However, we did not identify any particular influence from the inclusion of GPs trained in the TERM model; both positive and negative statements concerning the category of ‘multiple symptoms’ were disclosed by both trained and non-trained GPs. Furthermore, the quantitative analysis did not reveal any differences in the identification or classification of MUS between the two groups of GPs.

The insights gained in the early process of the analysis of the qualitative data were broadened and validated by consultation of scientific literature and inspired our use of the theoretical framework. Thus, the choice of framework derived from the data rather than was forced upon the data. The understanding of diagnosis as both a category and a process led to the identification of three themes of importance to the clinical usefulness of ‘multiple symptoms’. Different themes may have evolved if another theoretical lens had been applied. This does not per se compromise the internal validity of our results [20]. On the contrary, reflexivity in the process of generating and analysing data was ensured by interdisciplinary discussions taking place within the research group and from different epistemological perspectives, thereby fostering constant testing of and continuous reflection on the working hypothesis.

### Comparison with existing literature

The lack of consensus on categorisation practices found in the present study does not only relate to the category of ‘multiple symptoms’, but also to the classification of MUS in general [21,22]. GPs have previously been found reluctant to classify less well-defined disorders and, correspondingly, rarely apply a diagnosis of somatoform disorder [23-25]. Hence, the low prevalence of ‘multiple symptoms’ and the GPs’ lack of consensus on the contents of this category may not necessarily be a category shortcoming, but may rather reflect the difficulties embracing the application of contested diagnoses.

GPs have formerly been shown to construct their own criteria for patients with MUS based on e.g. unfavourable social background and problematic personality traits [26]. In line with these findings, GPs in our study applied subjective criteria of psychosocial and behavioural characteristics to distinguish patients with ‘multiple symptoms’ from patients with fear of a disease or with a somatoform disorder. Thus, the solely symptom-based criteria for ‘multiple symptoms’ diverged from the GPs’ clinical picture of patients with MUS; the latter reflecting more chronic and complex cases than ‘multiple symptoms’ was intended to capture. This was further illustrated by the fact that GPs labelled comparable proportions of patients with ‘multiple symptoms’ and somatoform disorder, although the category of ‘multiple symptoms’ was intended for less severe cases, which appear more frequently in primary care [27].

According to the GPs, time constraints were a barrier for addressing the perceived complexity of patients presenting with MUS. However, it may be questioned whether the barrier was, in fact, lack of time or rather uncertainty relating to the management of patients with MUS [28,29]. Consistent with previous findings, GPs in our study expressed a gap of uncertainty between recognition and management and had to use available – although inadequate strategies - for bridging this gap, e.g. avoiding the subject of MUS, addressing only single ‘valid’ symptoms in the consultation or offering less reasonable tests and referrals [30].

Continuity in the doctor-patient relationship is in general valued by GPs [31], and GPs in our study also found a continuous relationship to facilitate the recognition and management of patients with MUS. However, it has formerly been shown that relational continuity also holds the risk that GPs may comply with the patient and defy their own perceptions of adequate management in order to preserve a good relationship [30,32]. As investigation of the patient-doctor relationship was not the main scope of our study, we cannot say whether this issue was of great concern to the GPs. Instead, GPs were preoccupied with the stereotypical patient images that may be created over time and the pre-assumptions that may follow a diagnosis of ‘multiple symptoms’; both of these pose a risk of ignorance to symptoms and thus of overlooking serious disease.

In line with earlier qualitative findings for patients with depression [25], GPs in our study tended to use a biomedical frame of reference, thereby urging exclusion of all possible biomedical explanations before selecting the category of ‘multiple symptoms’. In other words, this category was used as a category of exclusion, thus equating it with specific diagnoses, e.g. somatoform disorders, rather than with other descriptive and provisional symptom diagnoses as originally intended. While GPs applied this symptom diagnosis as a specific diagnosis, they also considered it a risk indicator or a ‘yellow flag’, which could increase the awareness of patients with MUS and thereby help prevent iatrogenic harm and development of chronic disorders. While this needs further exploration in future studies, our results indicate that the category of ‘multiple symptoms’ may be better described and implemented as a risk indicator rather than a diagnostic category. Risk profiles have already been adopted in recent Dutch and German guidelines on the treatment and management of MUS [33,34].

## Conclusions

We face a challenge of identifying and caring for patients with mild-to-moderate conditions of MUS in order to prevent misclassification and iatrogenic harm. The present study of a proposed diagnostic category for these conditions demonstrates possible clinical benefits, such as increased GP awareness and informational continuity in partnership practices. However, the study points to a need for addressing GPs’ conceptual understandings of MUS and diagnosis if classification of mild-to-moderate conditions of MUS is to be applied in daily clinical practice. The described category of ‘multiple symptoms’ may profitably be used as a ‘yellow flag’ rather than a diagnostic category.

## Competing interests

MR is currently a Danish representative in Wonca’s International Classification Committee and a participant in the WHO’s Primary Care Consultation Group for the Revision of ICD-10 Mental and Behavioural Disorders. The other authors declare that they have no competing interests.

## Authors’ contributions

MR and PF initiated the study. MTR, FB, PF and MR designed the study. MTR collected, analysed and interpreted the data and drafted the manuscript. FB and MR contributed to data collection. RSA and MR contributed to the analysis and interpretation of qualitative data. All co-authors provided critical revision of the manuscript and approved the final version.

## Pre-publication history

The pre-publication history for this paper can be accessed here:

http://www.biomedcentral.com/1471-2296/15/118/prepub

## Supplementary Material

Additional file 1 GP registration form. Click here for file
