# Supplementary material for: Towards a clinically useful diagnosis for mild-to-moderate conditions of medically unexplained symptoms in general practice: a mixed methods study
Source: BMC Fam Pract. 2014 Jun 12;15:118. doi: 10.1186/1471-2296-15-118 (PMC4075929; doi:10.1186/1471-2296-15-118)
Supplement: Additional file 1 — GP registration form. [file 1471-2296-15-118-S1.pdf]

# GP registration form

Draft

## 1. Background information

Date: Day __ __ - Month __ __ - Year 2 0 0 9

The patient's civil registration number: __ __ __ __ __ __ - 0 0 0 __

Service offered: Tick only one box

☐ Service 0101 ☐ No service 0101
- the registration is finished if no service 0101

The patient is: Tick box if relevant

☐ Listed with another practice and is excluded
- the registration is finished

## 2. Problems presented

Problem(s) presented in the current consultation:

☐ No symptoms

☐ Symptoms which: Tick relevant boxes

| Cannot be attributed to a verifiable somatic disease or psychiatric disorder | Can be attributed to a verifiable somatic disease | Can be attributed to a verifiable psychiatric disorder (excl. P75) |
|---|---|---|
| ☐ | ☐ | ☐ |
| Please continue the registration ↓ | Diagnosis: ______ | Diagnosis: ______ |

## 3. Course

Type of consultation: Tick only one box

☐ New episode ☐ Recurrent episode ☐ Preplanned control

Course of symptoms: Tick only one box

☐ Single episode (=<6 months) ☐ Recurrent (=<6 months) ☐ Long-lasting (>6 months)

## 4. Symptoms

| Symptoms presented in the current consultation that cannot be attributed to a verifiable disease: | | Symptoms witin the past 6 months that have not been attributable to a verifiable disease: | |
|---|---|---|---|
| Specification: | Or ICPC code | Specification: | Or ICPC code |
| 1. | | 1. | |
| 2. | | 2. | |
| 3. | | 3. | |
| 4. | | 4. | |
| 5. | | 5. | |

## 5. Classification

Please tick the box below that best describes the patient's problem: Tick only one box

☐ A symptom diagnosis of 'multiple symptoms'
- at least 3 symptoms within the past 6 months
- the symptoms have not been attributable to a verifiable somatic disease or psychiatric disorder

☐ A symptom diagnosis of 'fear of a disease' (any A-Y chapter in the ICPC)
- the patient's problem is primarily fear of a disease and not the specific symptom

☐ Other symptom diagnoses (any A-Y chapter in the ICPC)
- single symptoms expected to resolve spontaneously
- symptoms in need of further assessment

☐ A somatoform disorder (P75)
- multiple, recurrent and changing somatic symptoms despite negative test results and doctor's reassurance
- symptom duration >6 months

G H I F __ __ __ P __ __ __
